# Informal payments and intra-household allocation of resources for health care in Albania

**DOI:** 10.1186/1472-6963-12-17

**Published:** 2012-01-23

**Authors:** Sonila Tomini, Wim Groot, Milena Pavlova

**Affiliations:** 1 Maastricht Graduate School of Governance, Maastricht University, Maastricht, The Netherlands; 2 Department of Health Services Research, CAPHRI, Maastricht University Medical Center, Faculty of Health, Medicine and Life Sciences, Maastricht University, The Netherlands; 3 Topinstitute Evidence-Based Education Research (TIER), Maastricht University, The Netherlands

**Keywords:** Intra-household, informal payments, nuclear household, extended household

## Abstract

**Background:**

Informal payments for health care services can impose financial hardship on households. Many studies have found that the position within the household can influence the decision on how much is spent on each household member. This study analyses the intra-household differences in spending on informal payments for health care services by comparing the resources allocated between household heads, spouses and children.

**Methods:**

Pooled data from two cross sectional surveys, the Albanian Living Standard Measurement Survey 2002 and 2005, are used to analyse both the probability and the amount paid in inpatient and outpatient health care services. A generalised Hausman specification test is used to compare the coefficients of probit and OLS models for nuclear and extended households.

**Results:**

We find that due to the widespread informal payments there are no significant differences between households in the incidence of informal payments for households' members, but there are more differences in the amount paid informally. Results suggest that households strategically allocate their resources on health care by favouring individuals with higher earning potential who have invested more in human capital. Extended households pay higher amounts for spouses with higher education compared to nuclear households. On the other hand, nuclear households choose to pay higher amounts for children with a higher level of education compared to extended households.

**Conclusions:**

The differences between households should be taken into account by public policies which should compensate this by redistribution mechanisms targeting disadvantaged groups. Governments should implement effective measures to deal with informal patient payments.

**JEL Codes: **I10, I19, D10

## Background

Health care expenditures are idiosyncratic risks imposed on households that can cause financial hardship, especially if the costs are uninsured. In many Central and Eastern European (CEE) countries and the Former Soviet Union (FSU) informal payments for health care are reported to be high and since they are informal, they are not covered by any formal insurance mechanism. The available strategies to cope with such risks can vary from digging into savings, selling assets, increasing labour supply, or reallocating resources within the household [1-3]. The position within the household and the number of household members may influence the decision on how much is spent on informal health care payments. Nuclear and wealthy households are believed to be better able to protect their children against higher expenditures associated with health care use than extended households. On the other hand, the amount allocated to adult household members may depend on their contribution to household income but also on the type of household [4].

This article looks at how different types of households respond to informal payments to medical staff when multiple household members use health care. We analyse the effect characteristics of household heads, spouses and children have on the probability or the amounts paid informally, and test for differences between these members of households. For this purpose, we use data from the Albanian Living Standards Measurement Survey 2002 and 2005. The article contributes to the existing literature on intra-household allocation of resources by providing insights on how households cope with unregulated payments in health care when the government policies fail and little is known about the actual prices.

The literature on informal payments has concentrated on the main causes and consequences of such payments [1,5-11]. Little is known on the impact that the decision-making within the household has on the amounts paid for different members of the household. The theory of social risk management [12] predicts that when governments fail to provide protection, the family and social network may help in coping with particular shocks. In the case of informal payments in health care, household members may support each other by redirecting financial assets or being involved in direct care during the hospitalisation periods.

In countries like Albania, where many households have preserved their patriarchal structure and gender inequalities are prevalent [13], it is important to see if decision making on health expenditure depends on the severity of illness or on the position that the patient has in the household. Such information is relevant not only to understand the dynamics of intra-household allocation of resources but also to guide policies that aim at redistributing income between household members. This can help to increase the effectiveness of programs like social cash transfers or health care subsidies to reach out to more household members (e.g. children or spouses).

### Informal patient payments in Albania

The high incidence of informal payments in most CEE and FSU countries is frequently attributed to the similarities in health care systems and the common difficulties in the transition from planned to market economies [11]. The ideology adopted by the communist regimes emphasised universal access to health care but did not focus on improving efficiency and quality of such services [14]. The fall of communism brought new challenges for these health care systems. The high number of hospital beds, overstaffed health facilities, underpaid health care professionals, and the reduction of the investments provided the conditions for informal payments [1,2,5].

The health sector in Albania has followed the same trends as other ex-communist countries where the health sector was underfinanced and considered a non-productive sector [15]. After the 1990s the public health care system is characterised by a low level of investments, a limited number of physicians (mainly concentrated in the big cities and the capital of the country), low quality of services, unqualified staff, unmotivated medical personnel, and frequent lack of medicines in public hospitals [15-17]. According to the Ministry of Health [18], in 2003 Albania spent around 5.9 per cent of its GDP on health and this has been more or less stable over the years. This level of spending is comparable to other countries in the region. However, only half of this amount is publicly financed and out-of-pocket payments remain considerably high.

Public health spending in Albania is financed by social health insurance contributions and general tax revenues. Health insurance in Albania should cover most of the formal costs of primary health care and all the costs of hospital care. Patients are formally required to pay small, fixed co-payments per visit in outpatient services or for specialised treatments in hospital care, but until 2005 the amounts required were very low. Payments or gifts made directly to medical staff are not foreseen in the financing of health care and episodes of 'under-the-table' payments requested by medical staff are considered as acts of bribery and punishable by law. However, a general lack of accountability and good governance combined with the lack of resources has contributed to a high incidence of informal payments in the public health sector [11]. Informal payments account for at least one quarter of hospital costs in 2005 [17], even though by law inpatient care is free of charge.

In the literature, informal payments are defined as direct cash/in-kind unofficial payments to health care providers and/or private purchases of health services and other products meant to be covered by the health system [19,20]. Country specific factors and data availability have a large influence on the use of this definition. Most of the previous articles in Albania based on LSMS (Living Standard Measurement Survey) data have identified informal payments as 'gifts paid to medical staff" [17]. The same definition is also employed in this article.^1^

The informal nature of such payments makes them unpredictable for individual households that find themselves exposed to the financial risks of health care events. Previous studies have shown that these payments directly influence the welfare of the household. Households may have to cope by using their savings, selling assets or borrowing money [1,21].

The informal nature and the dependence on health status and income rank informal payments among the most severe burdens imposed on individuals. In the absence of formal arrangements or a market for insurance against such risks (in Albania informal payments are also paid by a large share of individuals entitled to health insurance benefits) the burden is left to households and their social networks [12]. This refers mostly to support from other household members and to the coping strategies that the households employ on such occasions.

Previous research has shown that households facing catastrophic health care expenditures resort mostly to intra-household strategies for raising money to pay rather than to receiving help from extended households' members [2]. Households may decide to alter labour supply (i.e. if the household head falls ill other members may decide to work to substitute for the loss of incomes), sell property (or livestock in rural areas), or borrow money from relatives/banks [1,22,23]. The strategies employed by households may differ depending on the available resources, severity of the health impairment and treatment needed, the level of the payments demanded, or the household member in question.

There are various theories on how resources within the household are allocated over members. It has been shown that the share of contributed income determines the share of expenditures on each of the household members [24-26]. Other studies showed that female-headed households allocate resources more towards children, hence contributing to a better health status and higher enrolment rates [27,28].

We can view informal payments as an investment in health care. Medical care, together with education and training are investments in human capital [29]. The amounts invested in health care depend on the expected returns from such capital. Such expected returns are higher if health more critically depends on receiving health care, if remaining life expectancy is higher, if earnings are higher, etc. If we accept that informal payments help patients to obtain better treatment (and thus improve their health status) then we can assume that households will be more willing to allocate resources towards individuals with higher returns on health [30]. In this case, higher income earners would be favoured to invest upon i.e. households would be more willing to spend on women or spouses earning wages or in children as they have a longer remaining life expectancy than adults.

Households also differ in their decisions to spend on health care services. This may depend on the number of household members. Gary Becker [29] links the "quality" of children (the amount spent on each child) to the number of children in the household. Households tend to spend more on each child if the number of children is lower. This theory will also have direct consequences for health care expenditures and informal payments. It implies that households with more children and extended households will be less willing to pay higher amounts for health care on each child.

Households in Albania rely on their social network for getting the support that public policies cannot offer. Previous studies have demonstrated that family is the main source for borrowing money [31]. Households also rely to a greater extent on their social network for avoiding informal payments or improving the delivery and the quality of services [32]. Albanian social attitudes (especially in rural or deprived communities) tend to favour men. This creates gender inequalities and may also affect attitudes towards paying for health care. Such gender inequalities in Albania are reported in employment, health care, education and social life [13].

## Methods

The data used in this article come from the Albanian Living Standard Measurement Survey (ALSMS). The data are publicly available and anonymous.^2 ^For our analysis we pool data from 2002 and 2005 surveys. Both samples in these two cross sectional surveys are country representative. The households sampled numbered 3600 in 2002 and 3638 in 2005. The questionnaires include information on household demography, education, incomes, type of illness (sudden or chronic) and health care payments. In addition, information is available on the ways that households raise money to pay for health services. All the variables that we have included in our analysis come from identical questions in these two years.

The informal payments to medical staff include both in-kind gifts and in-cash payments (all converted to their monetary value). The number of individuals who have visited ambulatory services was 3583^3 ^while the individuals hospitalised numbered 1384^4 ^from a total of 31101 individuals in the sample. We have further divided households in two types: nuclear and extended. The division is based on the type of kinship relations between household members. Nuclear households include only two generation families. Household members here are the household head, the spouse and children (single headed households are also included in this category). The extended households include more than two generations, multiple younger couples living together, or couples living together with more distant relatives.

In order to investigate how individual characteristics influence informal payments to medical staff we use two different models for the probability of payments (the probit model) and amount paid (the Ordinary Least Squared model). The probability of paying informally can be considered as dependent on the individual characteristics of patients and other household members. Hence the probability of paying can be expressed as:

(1) $$P\left(y_{i}^{*}\right)=\left\{\begin{array}{cc} x_{i}\beta +\in_{i}, & if\;y_{i}>0\\ 0, & if\;y_{i}=0 \end{array}\right).$$

Where *y_i _*refers to the amount paid informally to medical staff, *x_i _*denotes a vector characteristics of individual patients and their household members, *β *represents a vector of estimated coefficients corresponding to such characteristics, and *ε_i _*is a vector of residuals errors that have a normal distribution.

Similarly, the amount paid informally to medical staff can also be considered as determined by individual characteristics of patients and their households. In such case the amount is expressed as:

(2) $$y_{i}=x_{i}\beta +\in_{i}.$$

This later equation is estimated using OLS. All models are run separately for nuclear and extended households. As our interest lies on testing for eventual differences between coefficients for these two types of households, we use a generalised Hausman specification test [33]. This post-estimation test allows us to compare coefficients in the models estimated for nuclear and extended households. The advantage of such specification test is that it makes use of the sandwich covariance estimator to adjust for any heteroskedasticities in the outcomes. Our models are estimated separately for inpatient and outpatient care. For comparative purposes, we follow the same procedure to estimate similar models for the total amount of out-of-pocket expenditures for health care.

## Results

### Descriptive statistics

Table 1 shows that about 26 per cent of the patients in outpatient care and 59 per cent of those using inpatient care have paid informally to medical staff. The incidence of payments varies little over nuclear or extended households.

**Table 1:** The incidence and amounts paid informally in outpatient and inpatient health care services.

	Outpatient Service		Inpatient Services	
**Household Types**	**The incidence of informal payments**	**Amount paid informally** **(per visit)**	**The incidence of informal payments**	**Amount paid informally** **(per admission)**

Nuclear household	0.27	104.78**	0.60	5203.23

Extended household	0.25	73.74**	0.58	4792.98

Mean for the total sample	0.26	91.25	0.59	5019.79

Note: Amounts are in Albanian Leks. All prices are deflated to 2002 prices. 100 ALL = 0.73 Euros in June 2002 (Bank of Albania, 2010). All series are deflated with 2002 prices. Stars indicate if the mean for the particular group is significantly different from the mean of all other groups (*** p < .01, ** p < .05, * p < .1).

However, nuclear households are more likely to pay informally for their members (both in outpatient and inpatient care) and at the same time pay higher amounts for household members in both services. Informal payments paid per admission in inpatient care are much higher compared to those paid for outpatient care. Patients pay approximately 55 times more per visit for inpatient than for outpatient care. This shows that such informal payments are in fact implicit payments imposed by medical staff.

Table 2 gives a detailed overview of the characteristics of children and spouses by household type. As we can observe, the education level of spouses in extended households is much lower than in nuclear households. Almost 71 per cent of the spouses in extended households have only primary education (8 or less years of education) and only 4 per cent of them have university education. Spouses in extended households are also older (with an average age of 48.85 years).

**Table 2:** Characteristics of spouses and children in nuclear and extended households

	Spouse of the household head			Children of the household head		
	**Nuclear Household**	**Extended Household**	**Total**	**Nuclear Household**	**Extended Household**	**Total**

Without education	0.00**	0.01**	0.01	0.05***	0.03***	0.05

Primary education	0.55***	0.71***	0.6	0.68***	0.63***	0.67

Secondary education	0.36***	0.24***	0.33	0.22***	0.28***	0.23

University education	0.08***	0.04***	0.07	0.05	0.05	0.05

Age	43.71***	48.85***	45.13	14.49***	20.50***	16.13

Has health insurance	0.43*	0.45*	0.43	0.33***	0.30***	0.32

Note: Stars indicate whether the mean for each household type is significantly different from the mean of the other type (*** p < .01, ** p < .05, * p < .1).

Children are also more likely to have primary education and health insurance in nuclear households. This may be due to the fact that children in nuclear households are on average younger compared to extended households. Therefore they are more likely to follow education and be covered by health insurance.^5 ^These first descriptive results show clearly that spouses and children in nuclear households seem to have invested more in human capital (they have on average more education). This may be due to a higher earning potential (as the data for spouses show).

Households use different methods to cope with uninsured health expenditures. Borrowing money is one of the most frequently used methods among them (see Table 3) attesting for the mutual help among household and community members (in the absence of formal mechanisms). The data show that nuclear households are more likely to borrow money compared to extended households. The next two popular methods are selling agriculture products or animals. These methods are more popular for extended households which live mostly in rural areas and rely more on products or animals as a form of insurance.

**Table 3:** Methods to raise money to pay for health care

	Nuclear families	Extended families	Mean of the total sample
Method to raise money -borrow money	0.653***	0.589***	0.626

Method to raise money - sell animal	0.176***	0.245***	0.205

Method to raise money - sell products	0.125***	0.181***	0.149

Method to raise money - sell valuables	0.009***	0.004***	0.007

Method to raise money - other methods	0.110**	0.097**	0.104

Note: Stars indicate whether the mean for each household type is significantly different from the mean of the other type (*** p < .01, ** p < .05, * p < .1).

### Results from Probit and OLS models

Table 4 gives the probit estimation results for the incidence of informal payments in outpatient and inpatient care as well as the generalised Hausman tests for differences between coefficients for nuclear and extended households. Results show that generally households seem more likely to pay for spouses than for household's heads (the reference category) and that this is statistically significant in extended households for outpatient care. On the other hand, paying informally for children is less likely than paying for household heads. This is especially the case for outpatient care (where coefficients for both nuclear and extended households are statistically significant) and to a lesser extent for inpatient care. This significant effect in outpatient care may be due to the specifics of illnesses for each household member as for example women needing more health care than men [34,35]. The differences in the probability of paying informally between two types of households are not statistically significant both for outpatient and inpatient care, suggesting that patients are faced with similar probabilities of paying for these services. The positive (but not significant) coefficient for children living in nuclear households and visiting inpatient care may suggest that whenever households face an increased risk of paying informal payments (descriptive statistics showed that the probability of paying is very high in inpatient care) nuclear households may be more willing to pay for their children.

**Table 4:** The Incidence of informal payments to medical staff in outpatient and inpatient health care services

	Outpatient Service					Inpatient Service				
	**Nuclear household**		**Extended household**		**Difference (nuclear- extended)**	**Nuclear household**		**Extended household**		**Difference (nuclear- extended)**

Spouse of the household	0.309	(0.344)	1.150**	(0.476)	-0.841	0.568	(0.499)	0.449	(0.503)	0.120

Children of the household	-0.589**	(0.288)	-0.329*	(0.193)	-0.261	0.168	(0.437)	-0.402	(0.301)	0.570

*(Household head)*										

Number of children into the household	-0.036	(0.026)	-0.053*	(0.032)	0.018	-0.019	(0.036)	0.041	(0.043)	-0.060

Sudden illness	0.106	(0.068)	0.198***	(0.076)	-0.092	-0.061	(0.112)	-0.243*	(0.125)	0.182

Year of the survey (year 2002 = 0; year 2005 = 1)	-0.255***	(0.069)	-0.222***	(0.081)	-0.033	-0.231**	(0.101)	0.024	(0.116)	-0.255*

Health insurance	-0.089	(0.073)	-0.267***	(0.082)	0.178	-0.299***	(0.105)	-0.270**	(0.120)	-0.029

ln income per capita	-0.006	(0.032)	-0.016	(0.036)	0.010	0.029	(0.045)	0.026	(0.046)	0.002

Living in rural area	0.174**	(0.075)	0.060	(0.085)	0.115	0.002	(0.109)	-0.059	(0.128)	0.061

Method to raise money - borrowing money	0.106	(0.065)	0.031	(0.076)	0.074	0.026	(0.096)	-0.138	(0.108)	0.164

Method to raise money - animal, products & valuable	0.083	(0.090)	0.124	(0.098)	-0.041	0.143	(0.132)	-0.024	(0.137)	0.167

*(Other method to raise money)*										

Age of the household head	-0.010**	(0.005)	-0.002	(0.002)	-0.008*	0.001	(0.007)	0.006**	(0.003)	-0.005

Age of the household spouse	-0.016***	(0.005)	-0.017**	(0.007)	0.001	-0.011*	(0.006)	-0.005	(0.008)	-0.006

Age of the household child	-0.004	(0.009)	0.011	(0.010)	-0.015	-0.003	(0.011)	0.007	(0.012)	-0.010

Gender of the household head	-0.133	(0.169)	0.142	(0.168)	-0.275	0.517*	(0.285)	-0.619*	(0.318)	1.136***

Gender of the household child	0.090	(0.112)	0.268	(0.201)	-0.179	0.142	(0.176)	0.497	(0.310)	-0.355

Level of education of the hh head^1^	-0.025	(0.036)	-0.024	(0.045)	-0.001	0.079	(0.058)	-0.123	(0.091)	0.203**

Level of education of the hh spouse	-0.003	(0.041)	-0.229**	(0.091)	0.227**	0.100	(0.062)	-0.041	(0.109)	0.141

Level of education of the hh child	0.201**	(0.084)	-0.160	(0.137)	0.360***	0.079	(0.107)	-0.006	(0.149)	0.086

Constant	-0.055	(0.393)	-0.319	(0.329)	0.263	-0.061	(0.567)	0.057	(0.398)	-0.118

Log likelihood	-1099.4186		-793.4290			-506.4827		-383.1305		

Pseudo R2	0.0396		0.0428			0.0301		0.0318		

Number of observations	1992		1504			765		575		

^1 ^Level of education is represented as a categorical variable where: (0) "Without education"; (1) "Primary education"; (2) " Secondary education"; (4) "University education"; (5) "Postgraduate education"

Notes: *** p < 0.01, ** p < 0.05, * p < 0.1. Standard errors and reference categories are in brackets.

The number of children in the household lowers the probability of paying informally to medical staff especially for outpatient care (statistically significant). The number of siblings in the household lowers the probability of paying informally for health care [36] and in addition children are more likely to be covered by health insurance. The effect is not as strong for inpatient care where the influence of health insurance is lower and informal payments are more spread. The nature of illness (having a sudden/acute condition) has a positive effect for both types of households in outpatient care. The results show that nuclear household patients with sudden illness have lower probability to pay informally compared with extended households. The nature of illness has the opposite effect for the inpatient care for both types of households.

The variable for the timing of the survey (2002/2005) shows that the incidence of informal payments has decreased for outpatient care for both types of households. However, this is not the case for inpatient care where informal payments have decreased for nuclear households but increased for the extended ones. The significant difference in coefficients, when the generalised Hausman test is applied, shows that when confronted with inpatient care such households are more likely to pay in 2005 than they were in 2002 and that this is definitely higher than for nuclear households. This can be due to the higher needs these households may have over time (e.g. having older household members who need extensive care).

Health insurance decreases the probability of paying informally both for nuclear and extended households in inpatient and outpatients care. This is encouraging, especially for inpatient care where the link between the contributions and service provision has been weaker. On the contrary, household income per capita does not have any significant effects indicating that informal payments affect patients throughout income distribution.^6^

The effect of the methods chosen by the household to raise money for health care on the incidence of informal payments does not vary between different types of households. However, a generalised Hausman test rejects the hypothesis of equal coefficients between nuclear and extended households for the method to raise money. The differences show that it is more difficult for extended households to raise money through borrowing or selling products or valuables. This indicates that they may use more often other ways for raising money like for example transfers from other relatives (even though the survey does not allow us to control for this).

Age of the patient has a negative effect on the probability of paying informally, except for extended households in inpatient care. This confirms our expectations about investments in health care for aging members of such households. A higher education of the patient has a negative effect on informal payments. But, as expected, the generalised Hausman test shows more differences between types of households (especially for outpatient care where effects are statistically significant). Mincer and Polacheck [30] argue that households invest more on members who will ensure higher returns in the future. Households choose to invest in those members with higher returns to human capital investments and children's payback time is longer and returns are higher [37]. In fact, a higher education of the child in nuclear households increases the probability of informal payments to medical staff suggesting that people in such households are willing to invest more in human capital (therefore they are more likely to pay for their higher educated children). On the other hand, spouses with higher education in extended households are less likely to pay informally especially for outpatient care. Having more education (and probably less resources) translates to more bargain power with medical staff. On the same time having higher education gives more chance to work in the formal market and to have health insurance.

Table 5 gives the OLS estimation results for the amount paid informally as well as the generalised Hausman tests for differences between coefficients for nuclear and extended households. The amounts paid informally in outpatient and inpatient care differ significantly from each other. The amounts paid for inpatient care are much higher (due to the severity of the conditions) and households have to prioritise the allocation of health expenditures based on the particular position of household members.

**Table 5:** The amount of informal payments to medical staff in outpatient and inpatient health care services

	Outpatient Service					Inpatient Service				
	**Nuclear household**		**Extended household**		**Difference (nuclear- extended)**	**Nuclear household**		**Extended household**		**Difference (nuclear- extended)**

Spouse of the household	0.278	(0.410)	0.362	(0.413)	-0.084	1.953***	(0.688)	-1.186*	(0.711)	3.139***

Children of the household	-0.214	(0.359)	-0.110	(0.198)	-0.104	-0.113	(0.620)	-0.460	(0.507)	0.347

*(Household head)*										

Number of children into the household	-0.066**	(0.029)	-0.045	(0.028)	-0.021	-0.153***	(0.051)	-0.188***	(0.061)	0.035

Sudden illness	-0.230***	(0.077)	-0.051	(0.069)	-0.179	-0.090	(0.162)	-0.415**	(0.200)	0.326

Year of the survey (year 2002 = 0; year 2005 = 1)	0.117	(0.079)	-0.005	(0.080)	0.122	0.119	(0.146)	-0.058	(0.178)	0.177

Health insurance	-0.170**	(0.079)	-0.226***	(0.076)	0.056	-0.277*	(0.153)	-0.161	(0.188)	-0.116

ln income per capita	-0.055	(0.034)	-0.001	(0.032)	-0.055	-0.014	(0.059)	0.088	(0.066)	-0.102

Living in rural area	0.227***	(0.084)	-0.005	(0.081)	0.231**	-0.129	(0.155)	0.054	(0.195)	-0.183

Method to raise money - borrowing money	-0.112	(0.074)	0.071	(0.071)	-0.184*	-0.144	(0.136)	-0.148	(0.167)	0.004

Method to raise money - animal, products & valuable	-0.113	(0.094)	0.251***	(0.086)	-0.365***	0.141	(0.182)	0.103	(0.212)	0.038

*(Other method to raise money)*										

Age of the household head	-0.007	(0.006)	0.005**	(0.002)	-0.011**	0.007	(0.010)	-0.011***	(0.004)	0.017*

Age of the household spouse	-0.008	(0.005)	-0.007	(0.007)	-0.001	-0.030***	(0.009)	0.001	(0.012)	-0.031**

Age of the household child	-0.001	(0.010)	-0.010	(0.011)	0.008	0.037**	(0.019)	0.017	(0.018)	0.020

Gender of the household head	-0.312	(0.202)	-0.037	(0.158)	-0.276	0.427	(0.360)	0.409	(0.566)	0.019

Gender of the household child	-0.136	(0.120)	0.213	(0.193)	-0.349*	0.097	(0.252)	0.160	(0.496)	-0.063

Level of education of the hh head^1^	0.099**	(0.045)	-0.107**	(0.044)	0.205***	0.132	(0.082)	0.253*	(0.144)	-0.121

Level of education of the hh spouse	0.044	(0.047)	0.164*	(0.093)	-0.120	0.077	(0.081)	0.569***	(0.163)	-0.492***

Level of education of the hh child	0.183**	(0.086)	0.269	(0.164)	-0.086	0.246	(0.166)	0.203	(0.239)	0.043

Constant	6.371***	(0.461)	5.501***	(0.296)	0.870	5.979***	(0.765)	6.058***	(0.569)	-0.079

R2	0.1092		0.1163			0.1170		0.1440		

Number of observations	521		361			438		316		

^1 ^Level of education is represented as a categorical variable where: (0) "Without education"; (1) "Primary education"; (2) " Secondary education"; (4) "University education"; (5) "Postgraduate education"

Notes: All series with 2002 prices. *** p < 0.01, ** p < 0.05, * p < 0.1. Standard errors and reference categories are in brackets.

Results show that in inpatient care nuclear households and extended households differ significantly in the amount paid informally for spouses (as compared to the household head and children). Nuclear households pay significantly higher amounts for spouses than extended households. The differences are also confirmed by the generalised Hausman test. Extended households are concentrated mostly in rural areas and the average age is higher than in nuclear households. This means that extended households are more exposed to health risks, and therefore they allocate fewer resources mostly to spouses. Even though they seem more likely to pay for spouses (see the right side of Table 4) they allocate on average fewer resources to them. Another reason could be the lower bargaining power that spouses may have in extended households (they are less educated and less likely to work).

The number of children is negatively associated with the amount paid informally in both nuclear and extended households. The effect is larger for extended households visiting inpatient care. This is consistent with what Behrman et al., [36] have found on investments on education. Households with many children are constrained to divide resources among a larger number of household members and therefore children with more siblings may receive fewer resources for health care than children with fewer siblings.

The coefficients indicating the years of the survey are not significant for neither the household types (contrary to what we observed in models for the incidence of paying) showing that informal payments have changed in numbers but not in substance (i.e. the amount paid). Health insurance appears to be important in lowering the amounts paid both for inpatient and outpatient care but the effect for extended households in inpatient care is lower than for nuclear ones. This may show that such households may have less bargaining power against medical staff.

The level of income per capita does not have any significant effect on the amount paid by both types of households. On the other hand, the generalised Hausman test indicates that methods to raise money for health care differ between types of households. When paying informally extended households are more likely to borrow or sell products, animals or valuables. This shows that shocks associated with health care events are more severe for these types of households. By affecting their income generating capabilities such events may have more long-term consequences for these households.

Age of the patient generally lowers the amount paid even though this effect may vary between types of households. For example, the Hausman tests show that older heads of extended households pay higher amounts for outpatient care but they pay lower amounts for inpatient care. Such change may be determined by the lower ability to pay for health care of the extended households.

Generally, households seem to be willing to pay more for males than for females and this may be again related to the theory of human capital (Albania is still a patriarchal society where males have more income generating power than females). Once more, education plays an important role in determining the amounts paid informally. Higher educated spouses in extended households pay significantly more than their counterparts in nuclear households. This indicates that education gives them more bargaining power within the household. Similar to the incidence of payments, nuclear households pay more for their higher educated children.

Table 6 presents estimates for the amounts paid out-of-pocket for inpatient and outpatient care. Out-of-pocket includes all the costs related to service fees, medicines, laboratory work, and transportation costs. In order to see the difference between out-of-pocket expenditures and informal payments, we have not included informal payments in the out-of-pocket payments. We use the same control variables in order to make results comparable with those on informal payments. The results confirm the trend for the total amount paid out-of-pocket in inpatient care for spouses and children. Households seem to pay more for spouses than they do for children (compared to the household head and other members of the households). This may be due to the better health insurance coverage of the children (paying less for formal fees or medicines) but also to the changes in specific health care needs.

**Table 6:** The amount paid out-of-pocket for outpatient and inpatient health care services (excluding informal payments)

	Outpatient service					Inpatient service				
	**Nuclear household**		**Extended household**		**Difference (nuclear- extended)**	**Nuclear household**		**Extended household**		**Difference (nuclear- extended)**

Spouse of the household	0.107	(0.252)	0.236	(0.324)	-0.129	-0.143	(0.577)	0.456	(0.580)	-0.599

Children of the household	-0.697***	(0.208)	-0.361***	(0.131)	-0.336	-0.100	(0.506)	-0.018	(0.341)	-0.081

*(Household head)*										

Number of children into the household	-0.016	(0.019)	0.022	(0.022)	-0.038	-0.095**	(0.042)	-0.020	(0.049)	-0.075

Sudden illness	-0.129***	(0.049)	-0.085	(0.054)	-0.044	0.166	(0.133)	-0.232	(0.147)	0.397**

Year of the survey (year 2002 = 0; year 2005 = 1)	-0.106**	(0.050)	-0.024	(0.057)	-0.082	0.094	(0.120)	0.096	(0.137)	-0.002

Health insurance	-0.253***	(0.053)	-0.065	(0.058)	-0.189***	0.006	(0.125)	0.072	(0.142)	-0.066

ln income per capita	0.011	(0.023)	0.014	(0.026)	-0.003	0.046	(0.056)	0.014	(0.056)	0.033

Living in rural area	0.199***	(0.055)	0.233***	(0.060)	-0.035	-0.023	(0.129)	-0.028	(0.149)	0.005

Method to raise money - borrowing money	0.206***	(0.047)	0.074	(0.054)	0.132*	-0.170	(0.112)	-0.081	(0.127)	-0.089

Method to raise money - animal, products & valuable	0.156**	(0.067)	0.222***	(0.070)	-0.066	0.008	(0.149)	-0.133	(0.158)	0.140

*(Other method to raise money)*										

Age of the household head	-0.008**	(0.003)	0.000	(0.001)	-0.009**	-0.006	(0.008)	0.000	(0.003)	-0.007

Age of the household spouse	-0.010***	(0.003)	-0.001	(0.005)	-0.009	-0.002	(0.007)	-0.009	(0.009)	0.007

Age of the household child	0.021***	(0.007)	0.022***	(0.007)	-0.001	0.020	(0.013)	-0.017	(0.014)	0.038**

Gender of the household head	0.034	(0.118)	0.096	(0.119)	-0.062	0.030	(0.324)	-0.586	(0.357)	0.616

Gender of the household child	-0.151*	(0.084)	-0.053	(0.140)	-0.098	0.029	(0.207)	-0.569	(0.351)	0.599

Level of education of the hh head^1^	0.048*	(0.026)	0.035	(0.032)	0.013	0.148**	(0.067)	-0.065	(0.101)	0.213

Level of education of the hh spouse	0.051*	(0.029)	0.061	(0.051)	-0.010	0.188**	(0.074)	-0.098	(0.143)	0.286*

Level of education of the hh child	-0.087	(0.064)	0.012	(0.084)	-0.099	0.006	(0.126)	0.416**	(0.172)	-0.411*

Constant	7.251***	(0.285)	6.428***	(0.240)	0.824	6.033***	(0.673)	6.379***	(0.480)	-0.346

						

Pseudo R2	0.1009		0.0660			0.0455		0.0345		

Number of observations	1871		1378			699		529		

^1 ^Level of education is represented as a categorical variable where: (0) "Without education"; (1) "Primary education"; (2) " Secondary education"; (4) "University education"; (5) "Postgraduate education".

Notes: *** p < 0.01, ** p < 0.05, * p < 0.1. Standard errors and reference categories are in brackets.

The results show that out-of-pocket payments have decreased throughout the years for outpatient but not for inpatient care. As we can see from the table, health insurance turns out to be not significant for the amounts paid for inpatient care. This is evidence for the smaller role that such mechanisms have in this service.

The Hausman tests indicate that nuclear households are again more able to borrow money for health care than the extended ones when paying informally for outpatient care (and thus confirming their higher borrowing abilities). However both types of households do not show a clear pattern when paying for inpatient care demonstrating that the level of out-of-pocket payments can be well beyond their immediate strategies for raising money.

The age of patients has a negative effect on the amount of out-of-pocket payments for the adults while the age of children has (mostly) a positive effect. It appears that households tend to spend more on out-of-pocket for older children than for the younger ones. This may also be related to older children's ability to generate an income and have a better position within the household in terms of resource distribution. Contrary to what we have found for informal payments, education of the spouse increases the amount of out-of-pocket paid for nuclear households. This change may reflect the complex composition of out-of-pocket payments (including fees, medicines, laboratory, and transport) showing that higher educated women in nuclear households spend more resources on higher quality services (e.g. by buying more expensive drugs or laboratory tests).

The nature of informal payments is different from that of total out-of-pocket payments and this may also contribute to slightly different results. However, the comparable findings for most of the variables suggest that informal payments are well embedded in the health care system of the country.

## Discussion

Our results do not show strong differences between household types in the probability of paying informally, indicating that informal payments are widespread and requested, and imposed on all households. Contrary to that, for the amounts paid there are differences between households in the way they allocate resources between their members. If we consider informal payments as an indicator of investment in health care, we can say that nuclear households appear to be more likely to invest in the health care of children and spouses. They spend higher amounts on children and spouses (compared to the other members of the households) than extended households. Spouses and children in such households are also likely to get more education and more likely to earn income from employment.

The findings generally support the hypotheses of health care as a human capital investment and give insight into how households in Albania react to informal payments in health care. We consider such informal payments an uninsured risk (as it is also often not entirely dependent on health insurance). In such situations households choose to allocate their resources over the individuals with the highest earning potential or the highest bargaining power within the household.

Nuclear and extended households differ in their decisions to allocate resources over their members in investing in medical care. The main factors relate to the specific position of the member (whether he/she is the head, the spouse, or the child) but also on other factors like age, education and gender. Our results show that households seem to be more likely to allocate resources to spouses than to children. As children in Albania are more likely to be covered by health insurance this may indicate a better protection against informal payments for children, and thus, less informal payments for them. The effect may also reflect the different type of health care needed for children and spouses. We find no significant differences between households in choosing to pay for different households members, but there are more differences when the amount paid is considered. The results show that for inpatient care nuclear households pay significantly higher amounts for spouses demonstrating that spouses may have better bargaining power in nuclear households. Descriptive statistics show that spouses in such households are on average older and better educated than in the extended households. They therefore may have higher income generating abilities and this may influence their bargaining power within the household. In fact, we show that education of the spouses influences significantly the amount paid informally in extended households (when compared to the nuclear ones).

The quality of human capital investments in children depends on the resources available, and resources available depend on the type of households. We show that both the incidence and the amount paid declines if households have a higher number of children. The education level of children seems to influence the decision on the amounts allocated for informal payments. Households choose to invest more on members that will ensure higher returns in the future [30] and children with higher education are the perfect example of this investment [37]. Individuals also choose to pay (invest) more on males knowing that they may generate higher returns (this is also given the patriarchal Albanian society).

In general, we find that patients in inpatient care seem to be more exposed to informal payments to medical staff. This could be due to limited health insurance for inpatient care and due to more severe health conditions treated. Higher payments and less protection from health insurance expose especially children and spouses to whom fewer resources are allocated. The positive effect that health insurance has on decreasing informal payments in outpatient care is encouraging. However, it is doubtful whether it may have the same effect in inpatient care where a more complex organisation and more resources are needed. Experiences from other former-socialist countries show that compared to tax-based funding, social health insurance may have a questionable social benefit [38-40]. This calls for more attention of policy makers in evaluating the social benefits (e.g. macro-efficiency and equity in the public health care sector) of introducing the health insurance in inpatient care in Albania.

## Conclusions

The findings of this study show that in absence of protection by formal mechanisms individuals with less bargaining power in the household (e.g. household members with lower education) are likely to get fewer resources to pay for informal payments to medical staff for health care. Extended households will tend to pay for members with more bargaining power and will invest less in children. Such differences should be taken into account by public policies which should compensate this by redistribution mechanisms targeting disadvantaged groups. Such mechanisms include for example, cash transfers conditioned on utilisation of health care by small children and young mothers, and subsidised health care for particular age groups.

The immediate coping strategies of households (i.e. borrowing money or selling products, animals or valuables) cannot mitigate the increased risk of large payments for health care and may also hamper their long-term income generating capabilities. This calls for more attention towards policies protecting the vulnerable households against uninsured risks. The intra-household allocation of resources for health care to children and spouses is certainly susceptible to the provision of such formal protection. The possible crowding-out effects of private transfers towards public transfers or social health insurance certainly remains a field for further research in the future. Also governments should implement effective measures for dealing with informal patient payments in general.

## Competing interests

The authors declare that they have no competing interests.

## Authors' contributions

ST designed and conceptualised the study, conducted the data analysis and wrote the first draft of the paper. WG contributed to the methodology and the finalisation of all the sections. MP contributed to the finalisation of all the sections. All authors approved the final manuscript.

## Endnotes

^1 ^Even though such definition does not fully capture all the informality in the health sector (e.g. payments for medicines or other materials that are otherwise provided free of charge), it clearly distinguishes the part of the out-of-pocket payments paid directly to medical staff. As the data show, such payments may be voluntary or requested but their incidence is high for both inpatient and outpatient.

^2 ^The full data sets and the accompanying documentation can be found at http://www.worldbank.org/lsms.

^3 ^From this total 2120 individuals have visited ambulatory services in 2002 and 1463 in 2005.

^4 ^From this total 707 individuals have been hospitalised in 2002 and 677 in 2005.

^5 ^In Albania children 0-7 years old and those following education are automatically covered by health insurance.

^6 ^Results using the total expenditures yield also similar results (available from the authors) but we have used household income to reflect better the income distribution.

## Pre-publication history

The pre-publication history for this paper can be accessed here:

http://www.biomedcentral.com/1472-6963/12/17/prepub
